# Imagination, ecologized and enacted: driven by the historicity of affordance competition

**DOI:** 10.3389/fpsyg.2024.1369820

**Published:** 2024-11-29

**Authors:** Caroline Stankozi

**Affiliations:** Institute for Philosophy II, Ruhr University Bochum, Bochum, Germany

**Keywords:** imagination, affordances, historicity, affordance competition, priming, synaptic plasticity, ecological psychology, enactivism

## Abstract

Together, ecological psychology and enactivism can explain imagination as being driven by affordance competition. This paper presents synaptic plasticity as a hotspot for the respective historicity. First, (i) affordances are introduced as directly perceptible on the ecological view, and as co-created by an individual on the enactive view. After pointing out their compatibility, (ii) empirical underpinnings of the historicity of affordance competition are summarized and followed by a non-representational interpretation thereof. They are used to explain: (iii) What affords imagining? After discussing both van Dijk and Rietveld’s in 2020 non-representational answer and McClelland’s in 2020 representational one, I propose a more general explanation: a stand-off between competing affordances can be resolved by imagination, driven by affordance competition. Arguably, (iv) the sensorimotor traces of previous interactions (e.g., strengthened synapses) can be repurposed as representations – grounding even representational explanations in an ecologized enactive framework.

## 1 Introduction

While cooking, you might imagine how cinnamon would go with your dish. What affords such an imagination? This paper argues that the answer is more interactive and driven more by automatic processes than it might seem. In particular, I will stress the role of your individual historicity: the affordances, possibilities for action, you have encountered before affect your sensitivity to current affordances. Their sensorimotor traces can even drive your imaginative explorations. Having cooked and tasted the dish before might have heightened your sensitivity to its taste, and how to balance it. Having used and consumed cinnamon in the past biases your expectations for how it would alter the taste.

Over the last decades, it has become clear that we should not think of agents essentially as brains in fleshy tanks. While cognitive processes are fascinating, all the magic does not happen inside a brain (as the sandwich view of cognition would have us believe, see Hurley, 1998). Nowadays, hardly anyone would deny that other parts of our body play at least some role in cognitive processes (be it via hormones, energy levels, or even our build). Many cognitive scientists have expanded the system they are concerned with from the brain to the whole organism – giving rise to interdisciplinary cooperations (Gallagher et al., 2013; Anderson and Chemero, 2017; Baluška and Levin, 2016). It is becoming increasingly common to further expand one’s inquiries to a situated organism-environment system (Baedke et al., 2021; De Haan, 2020; Favela and Chemero, 2016).

Tackling brain-centeredness has also been accompanied by attempts to bring forth non-representational explanations, especially for everyday interactions like sitting down on a chair. *Ecological psychology* (Gibson, 1979) for example argues that the environment is always patterned, and that we react to the patterns we perceive. No need to put representations of likely external sources together from sensory stimulation alone. *Enactivism* (Varela et al., 1991; Thompson, 2007) emphasizes the interactive nature of cognition, tying it more to interactive patterns in the organism-environment system than to brain-centered, decoupled representations. Chemero (2009, p. 154) acknowledges that enactivism should be integrated with ecological psychology to further improve the standing of a joint “radical embodied cognitive science.” Rietveld, Denys, and van Westen have even proposed an *ecological-enactive* framework (2018) – adding another “E” to *4E cognition* (as embodied, embedded, enactive, and extended). This paper is a step in that direction: Joining ideas from ecological psychology and enactivism to improve our understanding of an individual’s historicity and how it enables imagination.

Imagination is particularly interesting, since few people would disagree that, at least in abstract cases, it can involve representations. Arguably, this does not require us to postulate that organisms produce costly duplicates of everything they act on. Instead, I argue that even representational explanations benefit from being grounded in an ecologized enactive framework. Arguably, the sensorimotor traces left by previous interactions with affordances can be *used as* representations as soon as we become aware of them.

The following section (2) introduces the notion of affordances. Despite the direct perception stipulated by ecological psychology and the enactive focus on co-creating perception, some versions of both views are shown to be compatible. Then, (3) synaptic plasticity is presented as a hotspot for historicity, especially in affordance competition, followed by a discussion of non-representational interpretations of Cisek’s (2007) affordance competition hypothesis. The next section asks: (4) What affords imagining? Neither van Dijk and Rietveld’s (2020) non-representational answer, nor McClelland’s (2020) representational one offers a satisfying, general explanation of imagination. Combining resources from ecological psychology and enactivism, I propose an alternative: a stand-off between competing affordances can be resolved by imagination. Central enablers for this are synaptic plasticity, priming activities, and the resulting historicity. Finally, I argue that (5) sensorimotor traces of previous interactions can be repurposed as representations – grounding even representational explanations in an ecologized enactive framework.

## 2 Affordances

“Affordances” (Gibson, 1979) help us to refer to complex relations in the world more easily. This paper adopts Chemero’s view that affordances are relations between features of the *environment* and abilities of an *organism* (2009, p. 145). If you perceive a dish as seasonable and you are able to season it, it affords being seasoned by you – inviting you, say, to add some cinnamon. This relation is particularly tricky to spell out, since both relata can be broken down into further relations.

Chemero understands *abilities* themselves as relations between organisms and their environment. Abilities persist “because, at some point in the past, they helped the animal (or its ancestor) to survive, reproduce, or flourish in its environment.” (*ibid.*) As such, he sees abilities as functions, depending on evolutionary history (*ibid.*, p. 146).

Just as abilities are not of an isolated organism, *features* are not of the environment *per se*: Unlike perceptible properties of an object, features are related to a *situation* and are placed by an organism (Strawson, 1959; Smith, 1996; Chemero, 2001). The organism perceives that “the situation as a whole supports (perhaps demands) a certain kind of action” (Chemero, 2009, p. 140) – say, seasoning your pumpkin dish – and places a corresponding feature – it is *seasonable*: an affordance. This emphasizes the relational nature as well, since affordances are placed by the organism. They result from what *you*, with your individual abilities and needs, perceive *in* the environment.

Why do we pick up on the feature that our cooking of a pumpkin dish invites seasoning? Because it is relevant to us. Bruineberg et al. (2019, p. 5233) explain this with an intentional access to the world, provided by our skills – following Heidegger and Merleau-Ponty (they call it the Skilled Intentionality Framework; *ibid.*; see Rietveld and Kiverstein, 2014; Bruineberg and Rietveld, 2014; Van Dijk and Rietveld, 2017; Rietveld et al., 2018).

If skills enable us to place features, what are they? Through (assisted) learning, an agent acquires the ability to act appropriately in concrete situations (Rietveld, 2008, p. 976):

Once a skill is acquired, the relationship between body and world is modified. The individual is now attuned to a familiar environment. At that moment the level of ability rises to the point where the individual is able to perceive and respond unreflectively, yet adequately, to what Gibson called ‘affordances’ (Gibson, 1979, p. 127; Michaels, 2000).

In this sense, affordances are not tied to (actionable) properties of objects. The environmental part of affordances – which aspects of a situation stand out *to you* – is inherently *relational*. Whether a cinnamon container invites being picked up by you depends not only on your height, but also on the task you are currently engaged in and on whether you are in the habit of (unreflectively) using spices.

How do we perceive an action-inviting relation, though? Does our body directly pick up information from a dish about whether spices could be added to it? Or are we actively involved in sensing its seasonability? This section introduces and contrasts both ecologized enactive accounts of affordance perception.

### 2.1 Direct perception: picking up environmental information

Affordances were introduced to emphasize that the environment already structures and patterns stimuli richly, and that we can pick these patterns up directly instead of inferring them from scratch (Gibson, 1979). Gibson argues that perception does not require us to recreate the patterns and structures of our environment from individual threads of stimulation. Instead, he suggests that we pick up information from stimuli that are already patterned and structured as we encounter them. Why should we do the work of weaving representational patterns, if the environment already presents us with ready-made patterns?

Directly picking up information about the environment is not as tall an order as some might think. Especially representationalists might have a very demanding notion of ‘information’ in mind. For Gibson, though “*information about* something means only *specificity* *to* something” (J. J. Gibson, 1966, p. 187; original emphasis). The specific pattern of ambient light bouncing around in your kitchen is specific (i.e., univocally related) to that particular room and can in so far inform you about it (*ibid.*; Gibson and Gibson, 1955). The information can be described as a relation between the light and the environment it is bouncing around in (Chemero, 2009, p. 108). Note that direct perception does not equal passive perception: “Unlike stimuli, information is obtained by the agent and not passively imposed, and perception is a skill at which we can improve (Gibson, 1969; Gibson and Pick, 2000).” (Segundo-Ortin and Raja, 2024, p. 21).

Segundo-Ortin and Raja (*ibid.*, p. 24; added emphasis) note that “affordances are perceived within the context of *larger goal-oriented perception-action cycles*, with organisms striving to act adaptively in their ecological niche (Heft, 1989; Segundo-Ortin, 2020, 2022; Segundo-Ortin and Kalis, 2022).” They even mention the different goals of individuals, affecting whether they perceive a chair as an obstacle or as sittable.

Eleanor Gibson investigated how we learn to further develop our affordance perception. Her research program helps bring the individual and the skills she develops over time back into ecological psychology. As Adolph and Kretch, (2015) put it, she insisted that “we do not just see, we look. We do not just hear, we listen. Perceiving is an active process. The visual system, for example, is a motor system as well as a sensory one.” (Adolph and Kretch, 2015, p. 128) She even admits that in her earlier work, “the old mistake was to start with static displays in formulating a theory of perceptual learning” (Gibson, 1991, p. 615). She acknowledges the active nature of perception – as involving not only sensory, but also *motor* processes (Adolph and Kretch, 2015, p. 128) and not merely static stimuli, but *dynamic* events (Gibson, 1969, p. 16).

Chemero (2009, p. 155) gives an intriguing example for the skill of picking up information: simply by holding a book in your fingers, even with your eyes closed, you can tell how heavy, how long, and how thick the book is. You can tell whether it is vertical or angled towards the floor. This is because your muscles are actively holding that book, actively generating feedback from it with explorative movements. This is what is called “dynamic touch” (Gibson, 1962, 1966; Turvey, 1996).

### 2.2 Co-creating affordances

This closely resembles an essential enactive notion: *sense-making* (Varela et al., 1991, p. 149 f.). Thinking of sensing or perceiving as a chemical process at a single point in time does not do the complex interactive dynamics justice. Where Gibson highlighted the role of the environment, enactivists stress the role of an organism’s *ongoing* interaction with its environment. Through complex feedback loops, the present, past, and anticipated actions of an organism affect its sensing (the current position of your hand and your usual way of reaching for a drink affect which bottle in a pack you sense as most attractive). Thus, an organism does not simply sense an object or picks up information – rather, it co-creates^1^ what it senses. This is essentially what “sense-making” refers to (Thompson and Stapleton, 2009, p. 26; Di Paolo et al., 2010, p. 39; Froese and Di Paolo, 2011, p. 7).

Here, employing ‘sense-making’ can further emphasize that affordance perception is not a passive happening, but involves active doing on the part of the organism – even if there is no inference involved. It portrays affordance perception as an interactive process, in which the ongoing activity of the organism and the picked up, constraining environmental information *co-create* affordances.

The formulations – dynamic touch and sense-making – focus on different aspects. Gibson and his followers are especially interested in the flow of information. They focus on fairly abstract relations between parts of the (often inanimate) environment and a skilled organism. Enactivism centers on the concrete interdependency between an organism and its environment. The ecological view seems to describe the dynamic relations that exist within an agent-environment system more or less from a bird’s-eye view, featuring mathematical dynamical systems explanations in particular. The enactive view on the other hand describes more or less the same dynamics from within the organism, with a special interest in biological explanations. This contrast is not black and white – ecological research also deals with skills and how they are developed, while enactivists also investigate organism-environment dynamics. But they still employ quite a different set of vocabulary. Which explanation you should turn to in a specific situation does not only depend on how closely they fit your own line of thinking but also on the different aspects they highlight. By virtue of having different explanatory strengths which one provides a more suitable answer depends on your specific question. Chances are that you will be best off with a combination of insights from both. This is why I would like to help build bridges between them.

### 2.3 Ecological psychology vs. enactivism

The founders of enactivism, Varela et al. (1991, pp. 203f.), distanced themselves from an ecological approach. They see direct perception as incompatible with sensorimotor enactment, because they take the ecological environment to be a given. In Chemero’s (2009) variety, however, the informative invariants in the environment are *relations*. Since the organism is part of the environment-agent system, it can be one of the respective relata, e.g., when light reflects off its skin. As such, it co-creates what it senses. Chemero furthermore refers to features the organism places on a situation. The emphasis on its current interactions might not be as central as in enactivism, but skills play a decisive role in this. Since the training behind one’s skills is not far from the “histories of coupling” behind one’s sense-making Varela et al. refer to (Varela et al., 1991, pp. 203 f.), both views seem compatible after all. The perceived (version of the) environment is not independent of the organism in either view.

The only thing is that Chemero (2009, pp. 183f.) still holds onto – albeit neutral – *monism*: metaphysically, there is nothing but *experience*. Neither mental nor physical entities exist beyond that. Since perceiver and perceived are thus inherently entangled, one cannot explain one without (or even before) the other.

Let me describe what I make of this. First, independently of Chemero’s metaphysical view, experience seems to be relational: Experiences (calm, shock, silkiness, green-ness, seasonability) are subjective responses – or even resonances – to what one perceives: objects, events, or situations.

This paper does not hinge on any metaphysical view, but Chemero’s curious emphasis on experience, if understood as relational, befits the current purpose: emphasizing the *relational* nature of not only current affordances, but also of traces previous interactions have left in the way individuals interact with their world. If everything is experience, I take it that everything is relational. The relational nature of actions and situations is just what enactivists like Gallagher (2020, p. 7) want to add to typical ecological explanations.

While something relational can hardly exist without respective relata (say, situational features and agential skills), it is a metaphysical question which is the more basic thing: The relation or the relata. It is unusual to say that in essence, the relation is what everything is. It seems to be more common to claim that there is a situation and, somehow, agents have evolved in a way such that relations between them and their environment could arise (say, experience). But who is to say that the world/universe has ever been uninhabited? Regardless of which metaphysical position one takes, *relations* seem to be of central importance.

Disagreement on which came first–relata or relations–should not prevent fruitful exchanges on relational interactions. It does lead to different orders of investigation, though. Varela et al. (1991) contrast monism (like Chemero’s) with *dualism*: the view that the environment is pregiven and the organism as *representing* it or adapting to it (Varela et al., 1991, p. 202). Interestingly, they present enactivism as a *middle way* between monism and dualism: While there is an environment, the organism acts on its specific perspective on that environment. The *uniquely enacted environment* it is adapting to is thus co-created by itself. This results in a number of similarities with monism, since both its enacted environment and the organism itself are (partly) a result of its own activity (autopoiesis and sense-making – that is, roughly, creating itself and co-creating sensations). Thus, one could decide to investigate both together. But since enactivists acknowledge that there is a pregiven environment in the background, they tend to zoom in on the organism first, to then understand how it enacts its environment. Only then would they be ready to zoom out to the pregiven environment, by taking a look at wider dynamics.

In a nutshell, the ecological view starts with a “bird’s-eye view” on the agent-environment system. From that perspective, the environmental parts naturally dominate and draw most attention. The enactive view starts from the very beginning of the cell, noting how–and under which conditions–it becomes more and more complex. Its widening dynamics expand the scope of interest. Particular interactions or a wider social context of this organism can draw their attention to wider ecological dynamics.

While there is metaphysical disagreement, this does not *per se* prevent a fruitful exchange of data, or even collaborations. Especially given the mutual interest in organism-environment dynamics. Having two (albeit diverse) flocks of researchers working on roughly the same phenomenon from two different angles promises fast progress. Due to the different starting points and favored methodologies (referring to mathematical dynamical systems theory vs. biological findings), establishing ways to collaborate requires adjustments. But as both lines of research move closer to a medium perspective between the bird’s-eye view and that out of a developing cell, opportunities for collaboration develop more and more naturally, as evidenced by de Haan et al. (2013), Gallagher (2017, 2020), and Schlicht and Starzak (2021), among others. This paper proposes an ecologized enactive perspective on imagination, emphasizing the role of historicity in particular.

## 3 The historicity of affordance competition

Historicity goes far beyond the individual lifespan. Organisms have become attuned to certain environments over evolutionary timescales, so that every aspect of your body, say, the texture of your skin, or the general plasticity of your synapses, already carries an immeasurable historicity. Here, I focus on the traces an individual’s interactions add to her sensorimotor system.

Perceiving (co-created) affordances does not always lead directly to an action. Being faced with multiple action possibilities can result in what Cisek (2007) dubs *affordance competition*^2^. In the following, I will underscore that the competitors are not only sensorimotor activities invited by *currently* perceived affordances, but also traces of past interactions – weighted with regard to their previous success, among other things.

### 3.1 Priming: synaptic plasticity as a hotspot for historicity

One of the most fascinating features of neurons is their ability to be stimulated to varying degrees before they are activated. They do not simply have an on/off switch – they can be fine-tuned to the activities of countless other neurons. This phenomenon is known as *synaptic plasticity*.

The effects of synaptic plasticity go beyond the resulting local changes: taken together, making use of all that fine-tuning endows the network with emergent properties (Martin et al., 2000, p. 662; Morris, 1990). For example, its patterns become much more flexible, adjusting the behavior of the system even to subtle changes, e.g., in tasks, goals, or the environment. The strength of synaptic connections is based on our individual history. Say, you like the smell of the cinnamon you are adding so much that you want to taste more of it – you can then flexibly add more to your dish – possibly with some tasting, or even discussions, in between. If you are used to cooking alone, the seasoning process is less likely to invite verbalization. If you are used to cooking with others, your system might be more ready to ask, before adding more cinnamon than usual.

Through the inhibitory and excitatory relations between neurons (via the synapses), the activation of one neural pattern (e.g., for the perceived seasonability of your dish) creates an indentation into the activity of related patterns (e.g., affecting their responsiveness – priming you to reach out). And not just now: the complex interrelations between the patterns elongate the (indirect) effect one activated pattern has on the others (having ‘perceived the seasonability’ might prompt you to ‘comment on it’ while ‘dining’, respectively on ‘having seasoned it’ or ‘not’). This interrelatedness enables higher-order patterns to appear in the sensorimotor system as a whole: e.g., organizations of neural patterns – functional, temporal, spatial, or structural (Di Paolo et al., 2017, p. 183).

*Priming* can be understood as an umbrella term for both excitatory and inhibitory stimulation. However, a neuron is only activated (i.e., fires an action potential that primes other neurons – or motor regions) when a certain threshold is reached (which can take hundreds of stimulations). Due to such *high thresholds*, the first rounds of priming do not yet result in an action (Cisek and Kalaska, 2010, p. 281f.). Instead, the mutual excitation and inhibition result in a competition between patterns of neurons underlying mutually exclusive action patterns (*ibid.*; Cisek, 2007; Di Paolo et al., 2017, p. 199ff.). Elongating the process (through high thresholds) ensures that a wider variety of neural patterns can enter the competition in favor of a certain action. Beyond considering more possible actions, this results in a broader – and thus potentially more accurate – evaluation of the strongest competitors.^3^ You can think of this as enabling more (historically) relevant neurons to vote on which is to be activated next, instead of acting on the first couple of votes that enter.

### 3.2 Historicity without representations?

The underlying neural processes are traditionally thought of as representational. While this paper is not anti-representational, it aims to offer an alternative to traditional representational readings – especially for basic everyday cases below the height of abstract thinking.

When you are unsure whether to add cinnamon to your pumpkin dish, Cisek speaks of a competition between patterns of neurons underlying those mutually exclusive action patterns (Cisek and Kalaska, 2010, pp. 281f.; Cisek, 2007; Di Paolo et al., 2017, pp. 199ff.). In Cisek (2007), he related this to affordances. However, he described the competitors as “representations of potential actions” (p. 1586). While the idea is compelling, his phrasing seems unfortunate since Gibson proposed the theoretical concept of affordances as an *alternative* to the concept of representations (Gibson, 1979, p. 280).

More in line with non-representational accounts, Anderson amends that the competitors are “not representations of affordances but the *neural patterns* that are triggered by the perception of affordances” (Anderson, 2014, p. 219; added emphasis). He describes those neural patterns as implementations of different control systems, enabling the organism to enact invited actions (*ibid.*). In short, one could describe each contender as the *control knob* for a respective action: activating the control knob sets the action in motion. As such, Anderson suggests forgoing the focus on representations, instead linking the dynamically changing activation values in the brain to the sensorimotor control they execute (*ibid.*, p. 192). He understands pattern competition as reflecting “tension among various behavioral control loops that could be enacted” (*ibid.*, p. 203; p. 218), which he considers compatible with Cisek’s link between pattern competition and affordance competition (Cisek, 2007; Cisek and Kalaska, 2010).

However, this breaks with the idea of a resonating flow of information from the environment into action. Moreover, it postulates that some of the processes control others, where it is not strictly necessary to put it like that. Again, enactivism offers a dynamic, self-maintaining, activity-centered alternative.

As an equally non-representational alternative, the enactivists Di Paolo et al. (2017, pp. 198–200) call the competitors *sensorimotor schemes* (comprised of sensory, motor, and neural activities, *ibid.*, p. 61). The regularities within a sensorimotor scheme depend not only on various linked sensorimotor activities and a task, but also on relevant norms, e.g., speed, accuracy, or efficiency (*ibid.*). This description does not necessitate speaking of a control knob as such. Take the task of cooking a delicious pumpkin dish. Whether the emerging, habitual sensorimotor scheme turns out to include ‘adding cinnamon’ partly depends on the feedback ‘having added cinnamon’ generates: If the taste was improved or even generated compliments, this positively primes the cinnamon-adding scheme for further use – tying it more and more habitually to cooking pumpkin. This does not require any internal observer. The sensorimotor system rather stays constantly attuned to such feedback due to its structural features and activities – priming in particular. Its complexity brings historicity into any affordance competition. Being aware of part of the process is helpful, but optional (see Bruineberg and van den Herik, 2021, p. 4f.).

However, when it comes to what we call *mental* action, things are different. Many of them seem to be inherently tied to our awareness. Imagining how cinnamon would taste with your pumpkin dish is one such action. It is something you consciously do. Does this require the underlying process to be a different, more essentially representational one then? In the following, I will discuss a radical ecologized and a more representational explanation for imagination, to then offer an alternative.

## 4 What affords imagining?

Traditionally, affordances were seen as inviting *bodily* actions, like gripping, stepping or sitting. Recently, the debate has turned to the question of whether the environment can also afford *mental* actions, like imagination: imagining stepping on a slippery stone, or imagining how this cinnamon would go with one’s pumpkin dish.

Some have argued for radically embodied mental affordances (van Dijk and Rietveld, 2020; Kiverstein and Rietveld, 2021). Others have supplemented insights from ecological psychology with phenomenology and representationalism (McClelland, 2020; Jorba, 2020; for a critical discussion, see, respectively, Bruineberg and van den Herik, 2021; Segundo-Ortin and Heras-Escribano, 2023). Let us zoom in on a particular mental action: imagination. This section discusses whether and how imagination can be afforded by the environment.

### 4.1 Indeterminate unfolding of affordances across timescales invites imagining

Radical ecological psychologists offer a new perspective on imagination by telling a non-brain-centered story: putting into words how the unfolding of affordances over time can affect our actions (van Dijk and Rietveld, 2020, p. 5; Araújo et al., 2006, p. 661). Van Dijk and Rietveld (2020, pp. 4f.) point out that big projects like making an art installation involve both immediate small-scale affordances (picking up a phone) and larger-scale affordances (making the whole art installation) which unfold over several months.

They propose (*ibid.*, pp. 12 f.) that such a combination of affordances can invite describing certain processes as ‘imaginative’, under what I would summarize as three conditions: (i) Both small-scale and larger-scale affordances unfold over time. (ii) Their unfolding is indeterminate, i.e., it is unclear how those possibilities are going to unfold. And: (iii) The subject is skilled enough to sense when activities across different timescales are not unfolding in the same direction (e.g., the currently ensued large-scale affordance would enable visitors to walk through the finished art installation, whereas the currently installed cables might prevent them from doing so safely without tripping).

They essentially call an action *imaginative* when it is not yet clear how exactly it will turn out and how it would align with related, also still unfolding, affordances: “When affordances are conceived as possibilities that get determined in actual activity in real life situations, any engagement with affordances can be more or less imaginative depending on the determination achieved already.” (*ibid.*, p. 17) Action possibilities with such unclear results require more sensitivity to potential pitfalls, ideally even some double-checking whether they are pulling in a direction that is in sync with the overall project (*ibid.*).

In their example, the art installation essentially consists of carpets strung on cables in artistic patterns. In the beginning, the final number and positioning of carpets and cables was not yet clear. Still, the designers built a model at an early stage. Later, when they had to install another cable, the project leader opened a photograph of the old model on his phone. He pointed to it and told the others to imagine the newly installed cable “here” (*ibid.*, p. 16).

Van Diijk and Rietveld (*ibid.*) describe this as *continuing* a past activity (building the model) by *coordinating* with the photograph of the old model: “actively re-situating the (old) image further and further into the larger-scale process:” comparing the image to their current position, until they have actively achieved a “practical correspondence.” According to them, “[p]articipation in the large-scale process and the possibility to align the current activity with it again invited the use of the word ‘imagine’” (*ibid.*).

As such, they challenge the established representational picture. While I applaud their pioneering in introducing a more ecological way of describing even imaginative processes, their case of indeterminate unfolding *across* timescales seems to be a specific subset of imaginative processes. Having gained an idea of some situations that can invite imagination, the following two sections explore broader explanations that might tell us more about what lies at the heart of imagination.

### 4.2 Tricky affordances invite imagining

Various researchers try to incorporate affordance relations into their theories without buying into Gibson’s accompanying theory of direct perception. They describe affordances as being perceived indirectly. Meaning that the brain receives an incoming stimulus and then infers the likeliest source for it – actively creating a likely representation of the external event that might have caused that stimulation. One such representationalist is McClelland (2020).

As an excellent example for affordances for imagination, McClelland describes a situation where you want to cross a stream. There are stones in the water which afford being used as stepping stones. In the case of an easy route, you would simply go ahead and act on the steppability of those stones. However, if the route is a tricky one, he describes it as affording the mental act of rehearsing your actions in imagination (*ibid.*, p. 418).

The way he contrasts the affordances of the easy and the tricky route reveals how strongly he distinguishes bodily from mental actions. For him, they seem to belong to entirely different domains. Confronted with the easy route, he sees “no need to *infer* what kind of step can be performed” (*ibid.*). In relation to the tricky route, he does not explicitly refer to inference, but his description seems to imply that this is how he thinks of the “mental act […] of mentally rehearsing a viable route” (*ibid.*). He concludes that “[t]he space of perceptible affordances for bodily actions might even be *duplicated* in a space of perceptible affordances for the imaginative performance of those same bodily actions.” (*ibid.*, p. 419; added emphasis). This suggestion highlights the relatively strong divide he sees between bodily and mental actions.

Even granting this further dualist tendency, I doubt one must postulate that ‘mentally rehearsing step A’ requires an entirely different space of perceptible affordances from ‘taking step A’. This strong divide clashes with how closely stepping and rehearsing are intertwined in such a scenario. It seems more plausible that normally, the same affordance – stone A’s steppability – invites both kinds of actions. Do we not normally feel invited to step onto stone A – at least for a second – before mentally rehearsing how to go about it? This would indicate that the same, bodily, affordance underlies both the stepping and the rehearsal – at least initially. In referring to the second route as “tricky,” McClelland already indicates that there is something about the situation in addition to being a route that makes the difference for imagination. Let me explain how to think about this additional difference-maker, if not as a different affordance.

### 4.3 A stand-off between competing affordances can be resolved by imagination

Both van Dijk and Rietveld’s (2020) account and McClelland’s (2020) account can be helpful in explaining different instances and aspects of imagination. But the former only targets a subset of imaginative processes, while the latter seems ill-suited to explain instances of imagination which are as simple and situated as the given example (introducing divides where none seem necessary). With the interactive relationality of section 2 and the empirical underpinnings of historicity of section 3 in mind, I suggest the following interpretation of McClelland’s example (McClelland, 2020, p. 418):

The immediate affordance – a stone’s steppability – is the same both on the easy and the tricky route. It is the *context* that makes us hesitate: The stones themselves still invite us to step onto them. But the growing body of water in between adds increasingly strong affordances for falling into the water. Given that we are currently in a perception-action cycle that is tasked with bringing us to the other side dryly, the drownability of the water introduces a risk. Furthermore, if we previously experienced or witnessed a failed attempt at stepping on such a stone, or even a near failure, this primes/heightens our sensitivity to the slippability of the stone and/or the drownability of the water.

This can result in a stand-off between affordances, where the steppability and the slippability of a stone are equally strongly primed. Before this stand-off is resolved, it is unclear whether we will step on that stone or not. The delay and the unusually lively/costly back-and-forth between both options is likely to draw our *attention*. As such, reaching such a stand-off is likely to feel imaginative: becoming aware of the back-and-forth seems to be sufficient for basic instances of imagination. Conscious deliberation seems to be an optional add-on.

While the stone still invites us to step onto it, other (currently or previously) perceived affordances inhibit such a stepping movement. What we experience when “rehearsing” the tricky route is the *competition* between different action possibilities – which involves the activation of possible follow-up actions. While those competitors (say, ‘slipping’) were invited by the environment, they do not give us the whole story. The way our sensorimotor system (and further parts of our body) weighted and organized traces of past experiences affects how strongly a previous slip makes us hesitate to try again (Di Paolo et al., 2017, pp. 232ff.).

Arguably, the historicity of affordance competition (introduced in section 3.1) can help us understand imagination. In many cases, what feels like you imagining things in some mysterious, sophisticated way, might simply be you, becoming aware of some of the affordance competition that is steadily going on. Our *historicity* consists in traces left by previous interactions: If they caused a very strong inhibition, we will shy away from routes we deem similar, hardly even sparing them a second glance. If stepping onto those stones is not inhibited at all, we will not hesitate to follow even the tricky route. Only if there is enough inhibition to keep us from immediately executing the stepping, but not enough to make us turn away, are we indecisive. This is when affordance competition kicks in – and if we become aware of it (following its attentional pull), this suffices to experience imaginative scenarios–driven by the competing affordances and the way our sensorimotor system relates them to previous interactions.

Say, acting on one affordance (adding cinnamon to a dish) is inhibited by another affordance (saving your last pinch of cinnamon). Suppose the former invitation is still too strong to be fully disregarded and the latter inhibition only concerns your grasping movements. In this case, you might go on to at least imagine how the dish would taste with cinnamon. Previous encounters bias which direction your imagination is likely to take. If you normally enjoy the taste of cinnamon, you are likely to imagine it as making the current dish even tastier (potentially by resolving to get more afterwards). If you planned to use your remaining cinnamon for a special dish tomorrow, you might also imagine that (potentially doubting whether you can get it refilled in time). Depending on which priming is most effective, you might end up using your cinnamon now after all.

So, my claim is that a stand-off between competing affordances is always resolved by the same process: affordance competition. Whether we call it imagination seems to depend on whether we are aware of (part of) the process. In some instances of imagination, especially more deliberate, creative ones, the conscious part of our activities is likely to add some additional stimulations, possibly even fostering new connections. But what is the general driving force behind imaginative processes?

### 4.4 How traces of previous interactions drive imagination

Generally, a stand-off is resolved by (further) affordance competition. The widespread priming activities this requires are built into the high activation threshold of many of our synapses: since multiple priming activities are required, many *traces* of relevant previous interactions (in the form of strengthened connections, synaptic thresholds, priming relations, etc.) affect which synapses are particularly active now. If those underlying the ‘steppability’ are activated, we step on the stone, if the ‘slippability’ dominates, we refrain from doing so. The more unpleasant a previous experience, the more strongly will its traces now inhibit a repetition (i.e., the execution of the interaction which has preceded it in the past). The more one previously enjoyed the thrill of barely making it, the more will ‘stepping’ on a slippery stone be excited/primed. In a way, our system is checking how much weight it should give to the inhibitory signals from previous failed encounters with similar affordances: Did they previously cause enough damage to heed them?

While I do not see reasons to introduce a separate affordance for ‘mentally rehearsing step A’ in this scenario (unlike McClelland, 2020), one could introduce a mental affordance to compare stone A to a previously encountered one. Call it stone A’s ‘comparability’. Our interactive relation to the current environment invites *comparisons* to previous ones. Reaching a stand-off between competing affordances likely heightens our sensitivity to affordances for comparisons. To me, this is part of what imagination does. It takes patterns left from the past (e.g., in form of the weights of synapses, Werning and Cheng, 2017, p. 17) and checks whether they can be applied to the currently uncertain situation: Could they unfold in a similar way? Or, if they will not do so naturally: Can they be made to unfold in a similar fashion? In other words: Which relevant aspects are similar, which ones are different? Once those comparisons have started, they can of course go on in numerous ways. We can even go so far as patching together parts from different encounters to create an entirely new, more pleasant experience (like imagining a winged cat coming by and giving us a ride).

Unlike what van Dijk and Rietveld seem to suggest, to me, it does not seem necessary for the respective affordances to unfold *across* different timescales (which might be due to my linking of imagination to some instances of affordance competition, not to affordances as such). However, I agree that imagination does not normally seem to be invited by *one* affordance: it rather is a possible result of a stand-off during affordance competition. In so far, my account is compatible with their specific subset of imaginative scenarios: The affordances which clash in their case just stand in a particular relation to each other, one of indeterminate unfolding over time.

Imagination is not always about “to do or not to do X” but can also be about doing “p or q.” Conflicting affordances (taking the picturesque or the less steep route) can also result in a stand-off. There are different ways to resolve this. Another affordance might eventually outcompete both, for instance. But the complex, organized interconnectivity of your sensorimotor system has a fascinating way of collecting ‘votes’ for or against contending actions. The easiest explanation for *imagination-as-we-know-it* seems to be that if you become aware of this process, you feel imaginative (and rightly so). Imagination does not require any inferences – it is essentially what our sensorimotor system does anyways during *affordance competition*: A term that already communicates that this is not a conscious selection, but the result of a low-level process where previous interactions guide future ones. Proper imagination would simply require us to become aware of that.

In addition, you likely *can* influence the process consciously, but arguably, you do not *need* to. While you normally might – either by actively guiding your attention in a certain direction or by putting further constraints on the process – the competition would also proceed, and be resolved, without your conscious contribution. Arguably, you can also mentally ‘lean back’ and simply ‘observe’. Simply being aware of the competition is all it takes to experience imagination. But what about very creative, deliberate, or detached instances of imagination, which benefit from representational explanations?

## 5 Repurposing sensorimotor traces as representations

This section suggests that the traces of previous interactions can be used as representations thereof in conscious interventions. Making them (priming) traces first and potential representations second. This avoids the need for costly duplicates: Tying even representations more to what our resonance with our environment leaves us with.

### 5.1 One example, two explanations

Shepard (1984) also attempted to expand Gibson’s theory to imagination.^4^ Part of his approach is very sympathetic to mine – among other things, he mentions that there are “enduring but modifiable constraints that have been internalized through learning” or “through past experience by each individual” (*ibid.*, p. 432). While this sounds a lot like my *sensorimotor traces of past interactions*, there are important theoretical differences. They especially arise from the enactive ideas that have been developed since then–facilitating a more interactive story that shifts the focus away from a consciously experiencing individual to an effortless dynamic flow throughout the organism-environment system. While I also mostly zoom in on internal sensorimotor traces, I attempt to enactively link them back to previous interactions, whilst ecologically attributing them more to lasting effects of past affordances: tying internal changes back to long-term interactive dynamics throughout the organism-environment system.

Not equipped with enactivism, Shepard does not speak of sensorimotor traces, but of internal representations, or internalized constraints (*ibid.*, p. 422). He uses the circadian rhythm (which allows organisms to maintain a 24-h cycle) as an example for our “ability to take account of events with which we are not in physical interaction” (*ibid.*, p. 441). Here, I argue that this example can be explained without anyone “taking account.”

He particularly refers to hamsters who maintain a customary sleep–wake cycle in a lab with constant illumination and temperature (*ibid.*, p. 422; Elliott et al., 1972; Bünning, 1973). Over time, this leads to individual deviations from the 24-h cycle. Re-introducing concurrent lighting changes for all individuals re-aligns their 24-h cycles. How best to explain this?

Shepard’s circadian rhythm:

(A) Sleep–wake cycles match light–dark cycles (“the period of the earth’s rotation”).

(B) Mostly even when they are not directly coupled to the latter, e.g., when no lighting changes are perceptible.

(C) This indicates some internalized constraints, which represent light–dark cycles (“the period of the earth’s rotation”).

While constant illumination leads to increasing individual differences, they are resolved once changes in lighting are re-introduced, by recalibrating the internalized constraints. Interestingly, the match between internal and external constraints is attributed to hamsters having evolved in this world with its particular period of rotation – not to individual efforts (*ibid.*, p. 422). This makes for a very sleek explanation. However, is it necessary to invoke representations to explain something as automatic and basic as a circadian rhythm?

Ecologized enactive alternative:

(A*) Sleep–wake cycles usually involve interactions with light–dark cycles.

(B*) Actions previously afforded by (features of) light–dark cycles keep being performed even after the latter have not been directly perceptible.

(C*) This indicates that a steady, loopy repetition of sensorimotor patterns/perception-action cycles can be guided by traces of previous interactions (say, strengthened synaptic connections between the sensorimotor schemes of ‘sleeping for about X breaths’ and ‘taking about X bites’, adjusted hormonal cycles, strengthened muscles, or even by keeping snacks nearby when sleeping).

This holds even if formerly action-guiding lighting changes are not currently perceptible. The actions they (previously) afforded are tied to other sensorimotor patterns by sensorimotor traces. This transpires over individual or evolutionary timescales, creating something you can metaphorically think of as a ‘force of habit’. Therefore, the usual actions keep being activated even in constant lighting. Arguably, just before the onset of those formerly afforded actions, the organism remains particularly sensitive to lighting changes (due to the strengthened synapses). Re-introduced lighting changes can thus turn habits of ‘formerly afforded actions’ once again into ‘directly afforded actions.’ (Equaling out the individual temporal differences resulting from non-uniform biting speed, environmental interferences, etc.)

Here, instead of representations, I find it more befitting to speak of (i) habitual (or even innate) *sensitivities* located within perception-action cycles, and (ii) habitually encountered *affordances* guiding the resulting actions. Should those affordances not arise as usual, traces from previous encounters (which one can think of as ‘force of habit’) can keep the habitual behavior up, if they are ingrained deeply enough. In so far, I agree with Shepard that there are some crucial constraints that allow for the persistence of the sleep–wake cycle. But instead of linking them to (representations of) the light–dark cycle, I link them to (traces of) previous sleep–wake cycles *invited by* previous light–dark cycles (i.e., not to objects or external events, but to previous interactions with them).

### 5.2 Traces that automatically prime sensorimotor activities first

The hamster example does not seem to require being explained as *imaginative*. It seems unnecessary to claim “that animals have internalized the invariant period of the earth’s rotation.” (Shepard, 1984, p. 442) While the earth’s rotation is a central cause for bringing the respective traces about, I do not see why animals should abstractly represent its “possible projections and transformations” (*ibid.*). Instead, I favor an *action-centered* explanation. What they rather seem to have gotten used to is *their* typical fluctuation between sleeping and being awake–which has established itself through repetition (be it over evolutionary or individual timescales). Those traces are not predominantly internal ones: They also include the snacks a hamster keeps beside its sleeping spot, for instance–inviting eating as soon as it awakes. Further traces of previous sleep–wake cycles include hormonal cycles, or the tuned excitability of relevant neurons at certain points in the cycle (say, priming the hamster to curl up for the usual duration after eating).

Even the internal ones among the traces I am concerned with are not constraints regarding (abstractions of) an *object*, but traces of *situated interactions*. Shepard, on the other hand, has events like the earth’s rotation in mind. Even if the respective constraints are acquired over the course of evolution, an “ability to take account of events” (*ibid.*, p. 441) would add an additional meta-level which does not seem strictly necessary. Should such constraints be empirically identified, it seems more befitting to describe them as *resonating with* constancies in the environment, rather than representing them (Raja, 2018). While an observer can see them as representations, they are not *used as* stand-ins: While they might have a similar effect as light–dark cycles, they do so without being used *by* anyone.

Hutto and Myin (2013) influentially argued that sensitivity to, say, cinnamon, normally comes without a label for the organism: It is one thing to be sensitive to cinnamon, and quite another to be sensitive to descriptions of it. Even if one does not see all representations as “descriptions,” the central difference between a low-level process and some form of a meta-process (involving content) remains.^5^ Regardless of one’s take on representations, at least some cases can be explained with priming activities and habitual interactions with (previously encountered) affordances alone, without the need to invoke representations. An organism’s natural historicity can make representational explanations superfluous.

One could call the sensorimotor traces I am talking about representations of lighting changes. But representational *for whom*? Only for an observer. To me, representational explanations are more interesting when the organism itself uses sensorimotor traces *as* representations for the interactions they arose from: Something beyond the low-level activities of the respective sensorimotor patterns.

### 5.3 Traces that are used as representations second

A representational story becomes more relevant when perceptible environmental information is reduced (e.g., through fog). Our activities can then be supplemented by “whatever constraints operate within” (Shepard, 1984, p. 422). In the hamster example, it did not seem advisable to describe those constraints as being used *as representations*.

But what about the pumpkin dish? Assume that you have just brushed your teeth and can thus not reliably taste it yet. The smell nonetheless invites you to add a hint of cinnamon. If this would be a new combination for you, fear of ruining the flavor might keep you from acting on this affordance. In this stand-off, your sensorimotor priming activities kick in and ‘vote’ for or against adding cinnamon (called ‘affordance competition’). Since your taste buds are still affected by your toothpaste, you rely on traces of previous relevant interactions–not just of tasting food, but also of hearing praise for unique combinations, of reaching for spices, or even for a glass of water to get rid of an unpleasant taste. As soon as you are ‘listening in’ on the ongoing sensorimotor competition, you are using it *as representations* for previous/possible interactions and the encouraging or discouraging connections between them: You are *imagining* how well cinnamon would go with your pumpkin dish – driven by sensorimotor competition.

McClelland (2020, p. 408) explained affordance competition as generally presenting the conscious individual with a menu of options to choose from. The usual, ongoing competition, however–including its resolution–is best explained via automatic priming activities (see Bruineberg and van den Herik, 2021, pp. 4f.; Cisek and Kalaska, 2010, p. 275). Doing that turns any additional conscious deliberation into an optional add-on, applying its explanatory repertoire only to the cases where it is needed. Proper instances of imagination might be such cases, depending on one’s theory of consciousness.^6^

How to account for abstractness? Traces of the most relevant past interactions compete for activation–finding the most suitable one(s) for the situation we are currently interacting with. Notably, only (overlapping) parts of them might be activated (say, those that are shared by multiple instances of tasting cinnamon-flavored dishes). If we become aware of them, this can render the resulting imagination fairly abstract, providing us with an abstract version of what cinnamon can add to a dish. This is facilitated by respective higher-order patterns, which can be functional, temporal, spatial, or structural (Di Paolo et al., 2017, p. 183).

It is crucial to me that individuals do not create sensorimotor patterns or even traces in a mostly decoupled, inference-driven state. Rather, interactions naturally leave those action-guiding traces – an organism cannot help being affected by repetition (habituation, for example, can be found even in bacteria: if a stimulus persists, this eventually leads to a decreased response to it; Tang and Marshall, 2018; Wood, 1969). Our sensitivities and abilities allow interactions to take place and prime us, but it is the environment-agent system which is doing the priming–not some internal observer, our genes, or the brain. Since sensorimotor traces are left by previous encounters, the organism was actively involved in creating them. But the underlying dynamics are interactive and relational: they result from a more and more finely tuned resonance to environmental situations and the task at hand.

I see the, respectively, emerging sensorimotor patterns – or rather schemes – (‘cooking pumpkin’ habitually leading to ‘adding cinnamon’) roughly like tree rings: They *can* be seen as representing the respective interaction, or even more abstractly as part of our interactive history – but they are not normally used as such. Grounding even representational explanations for imagination in an ecologized enactive framework enables us to view representations not as indirectly produced by decoupled observers, but as traces that are naturally left by interactions with the environment. Those traces can be repurposed as representations for the respective interactions.

At least many human animals are capable of some meta-processing: They can become aware of having a certain skill, or of wanting it, and can think about how best to acquire it. But importantly, this is an additional, optional activity. While it discloses a whole range of wonderful activities (discussing hopes and dreams, understanding our interactions better, and passing on the complex skill set of a surgeon or an engineer), this meta-processing is not necessary for most skillful interactions. Becoming sensitive to our sensorimotor patterns, we can use them, as we use tree rings to infer the age of a tree. But even if we never became aware of those patterns, and the connections between them, they would still do their job. As such, I argue that previous interactions leave traces which automatically affect sensorimotor priming activities first, and can be used as potential representations second. The historicity they add to our sensorimotor activities is what drives our imagination.

## 6 Conclusion

This paper presented imagination as driven by the common, not normally subjectively controlled, process of affordance competition. In a stand-off between conflicting action possibilities, becoming aware of the resulting sensorimotor priming activities can feel imaginative. It is likely that one can consciously affect the process, e.g., further supporting one competitor, thinking of another alternative, or even creatively combining parts of previous interactions. But this is *not necessary*. Even if we only become aware of the affordance competition, without consciously influencing it, we still experience imagination, since the priming activities go on anyways.

Insights from ecological psychology and enactivism were connected to derive a general understanding of imagination. This general, ecologized enactive explanation seems to be compatible with van Dijk and Rietveld’s non-representational account (2020). The subset of potentially conflicting affordances they have in mind are ones which unfold indeterminately across timescales. Where they emphasize the environmental invitations, I add details on the traces they leave in the sensorimotor system. In contrast to McClelland (2020), my view furthermore offers a less costly and less dualistic way of deriving representations: explaining them as repurposed sensorimotor traces of previous interactions. Notably, many complex interactions can be explained with low-level sensorimotor activities alone, without requiring the organism to use them as representations. But even in cases of imaginative explorations, the required historicity is largely enabled by synaptic plasticity. This allows traces from long-gone interactions (say, strengthened synapses) to guide our current interactions, by affecting the continuously ongoing competition between possible afforded actions. Becoming aware of this competition, and potentially even consciously influencing it, is what can be experienced as imaginative.

## Footnotes

^1^This acknowledges the individual’s active role in picking up and resonating with its unique range of environmental information. Unlike creating representations, this does not require inferences or actively replicating anything.

^2^While the notion is misleading, I keep the label for the purpose of this paper, since it does link the competition back to the initial affordances. What is competing though are not affordances, but invited sensorimotor activities (see McClelland, 2020, p. 409, fn. 4; Bruineberg and van den Herik, 2021, p. 5, fn. 3 – while they speak of *motor* activities, I am less inclined to disentangle them from their intricate sensorimotor feedback loops).

^3^This does not pertain to situations which elicit prompt reflexes. Repetition can similarly prevent any real competition, prompting the usual response by force of habit – potentially even creating automatisms (Di Paolo et al., 2017, pp. 145f.).

^4^I am grateful to one of the reviewers for pointing out the relevance of Shepard’s work.

^5^Glock (2019) makes a similar distinction when arguing that *acting on* a reason does not require being consciously *aware of* it. Combined with Starzak and Schlicht’s (2023) suggestion that affordances can be understood as reasons, this indicates that acting on affordances does not imply being aware of them.

^6^Radical enactivists or even Gibsonians might tend to offload imaginary processes into the environment. This would require more assumptions, though, and a detailed account of the underlying mechanisms. Instead, this section indicates how an ecologized enactive understanding can improve even representational explanations.
